# Enhanced cobalamin biosynthesis in *Ensifer adhaerens* by regulation of key genes with gradient promoters

**DOI:** 10.1016/j.synbio.2022.04.012

**Published:** 2022-05-04

**Authors:** Sha Xu, Zhiqiang Xiao, Shiqin Yu, Weizhu Zeng, Yongming Zhu, Jingwen Zhou

**Affiliations:** a National Engineering Laboratory for Cereal Fermentation Technology, Jiangnan University, 1800 Lihu Road, Wuxi, Jiangsu, 214122, China; b School of Biotechnology and Key Laboratory of Industrial Biotechnology, Ministry of Education, Jiangnan University, 1800 Lihu Road, Wuxi, Jiangsu, 214122, China; c Science Center for Future Foods, Jiangnan University, 1800 Lihu Road, Wuxi, Jiangsu, 214122, China; d Jiangsu Provisional Research Center for Bioactive Product Processing Technology, Jiangnan University, 1800 Lihu Road, Wuxi, Jiangsu, 214122, China; e Hunan Hongying Biotechnology Co. Ltd., 10 Hongying Road, Jinshi, Hunan, 415400, China

**Keywords:** *Ensifer adhaerens*, Cobalamin, Genome sequence, Transcriptome sequence, Promoter, Overexpression, Combinations

## Abstract

Cobalamin is an essential human vitamin widely used in the pharmaceutical, food, and feed additive industries and currently produced by bacteria or archaea. *Ensifer adhaerens* HY-1 is an industrial strain that also produces cobalamin. However production outputs are poor and the specific synthesis pathways require characterization. In this study, the whole genome sequence of *E. adhaerens* HY-1 was generated and annotated, and genes associated with cobalamin biosynthesis were identified. Then, three genes, *CobSV*, *CobQ*, and *CobW* were identified as the most efficient ones for enhancing cobalamin synthesis. By transcriptome sequencing of *E. adhaerens* HY-1 cells at different growth stages, 65 endogenous promoters with different gradient strengths were identified. After combined expression of different strength promoters and key genes, a high cobalamin-producing recombinant strain, ‘*hmm’* (genotype: P_*metH*_-*CobSV*-P_*ibpA*_-*CobQ*-P_*mdh*_-*CobW*), was generated. Cobalamin production was 143.8 mg/L in shaking flasks, which was 41.0% higher than the original strain. Cobalamin production was further enhanced to 171.2 mg/L using fed-batch fermentation. Importantly, our data and novel approach provide important references for the analysis of cobalamin synthesis and other metabolites in complex metabolic pathways.

## 1 Introduction

Cobalamin (also known as VB_12_) is an essential vitamin for maintaining normal growth and function in the body. It participates as a coenzyme in various metabolic processes where it promotes the transfer of methyl groups, promotes cell maturation, and participates in the isomerization of specific compounds [1]. Recently, the vitamin has attracted considerable attention because of increasing demands. The cobalamin production with current synthesis method remains under-supplied, causing high market prices [2]. Therefore, improvements in industrial production outputs are warranted. Cobalamin belongs to a class of porphyrin compounds that contain a corrin ring [3], and it is one of the largest non-polymeric natural compounds [4]. It prevents anemia and senile dementia, and is widely used in pharmaceutical, food, and feed additive sectors [5]. Currently, industrial cobalamin production is primarily conducted via microbial fermentation routes [6], but these production outputs require considerable improvement [7].

Cobalamin is a typical porphyrin compound [8] and highly complex vitamin, the structure of which contains a porphyrin ring connected to a cobalt ion to form a central skeleton structure [9]. Due to its complexity, chemical synthesis processes require more than 70 reaction steps making industrial production particularly difficult [10]. Previous studies have reported that certain bacteria and archaea synthesize cobalamin [11]. Currently, it is produced via microbial fermentation using *Pseudomonas denitrificans*, *Ensifer adhaerens*, and *Propionibacterium freudenreichii* [12]. Most production enhancing strategies have focused on fermentation optimization processes and the mutation breeding of strains [13]. However, few reports have elucidated the regulation and optimization of cobalamin metabolic pathways.

**Fig. 1:**
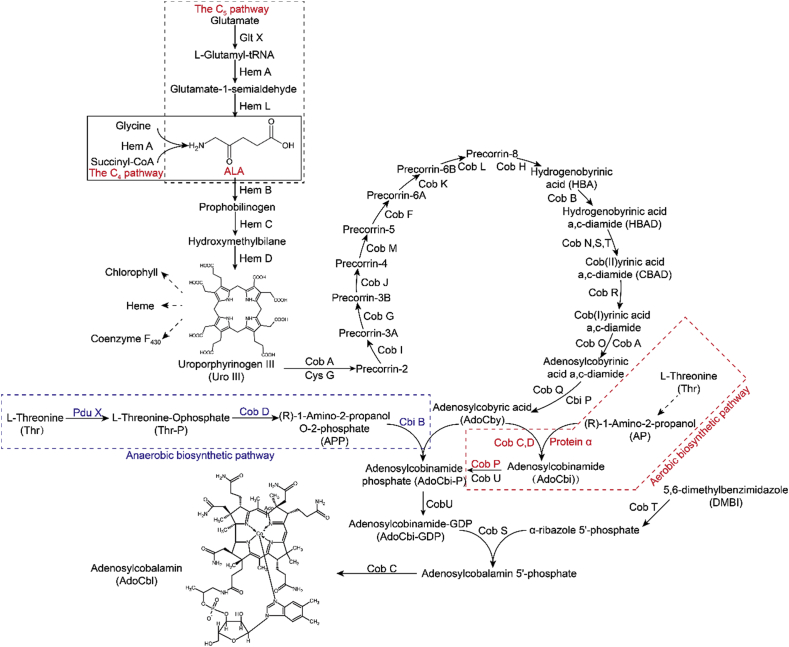
**The cobalamin biosynthetic pathway in microorganisms. (A)** 5-aminolevulinic acid (ALA) synthesis is divided into C4 and C5 pathways according to different sources. The intermediate product, uroporphyrinogen III (Uro III) branches to produce porphyrins, such as chlorophyll and heme. Differences between aerobic and anaerobic pathways are primarily characterized by the insertion time of Co^2+^ and oxygen. The red dotted box showed the aerobic biosynthetic pathway and the blue dotted box showed the anaerobic biosynthetic pathway.

The synthesis of cobalamin requires the formation of porphyrin ring, which is mainly divided into two forms [14]: one is based on the substrates, glycine and succinyl-CoA which generate the skeleton, uroporphyrinogen III (C4 pathway) [15], whereas the other uses glutamic acid to synthesize the skeleton, uroporphyrinogen III (C5 pathway) [16]. Uroporphyrinogen III then generates cobalamin under the action of the *Cob* (aerobic pathway) or *Cbi* (anaerobic pathway) series of genes [17] (Fig. 1). The entire *de novo* synthesis pathway requires the participation of more than 30 genes [18], with genes subjected to feedback regulation [19]. In addition, the dominant cobalamin production strains are all non-engineered, as reflected in previous studies where complexities/difficulties were encountered when genetically manipulating cobalamin metabolic pathways [20,21]. In the model strain, *Escherichia coli*, a *de novo* cobalamin synthesis pathway was constructed and cobalamin production was optimized to 307.0 μg/g DCW [17].

*E. adhaerens*, a recognized cobalamin industrial production strain, generates the vitamin with aerobic pathways [22]. In this study, an industrial strain, *E. adhaerens* HY-1 was investigated, and genes related to cobalamin synthesis were analyzed by genome sequencing. The genes related to cobalamin synthesis were overexpressed in pBBR1MCS-5 plasmid with P_*dnak*_ as the promoter. Using this strategy, three key genes—*CobSV*, *CobQ*, and *CobW*—were identified. Based on gene transcription analyses at different growth times, 65 promoters were selected. Then, promoter expression strength was verified using the reporter gene, *mCherry*. Finally, via combinatorial overexpression of different strength promoters and genes, a recombinant strain (genotype: P_*metH*_-*CobSV*-P_*ibpA*_-*CobQ*-P_*mdh*_-*CobW*) increased the production cobalamin by 41% compared to the original strain. Cobalamin production was further enhanced by a fed-batch fermentation. Our study provides valuable insights on the enhancement of cobalamin synthesis via metabolic engineering and the comprehensive dissection of complex metabolic pathways for the production of other key metabolites.

## 2 Materials and methods

### 2.1 Strains and plasmids

Engineered strains were constructed using *E. adhaerens* HY-1 supplied by Hunan Hongying Biological Technology Co., Ltd (Jinshi, Hunan, China). Strain names and genotypes are shown (Table S1). *E. coli* JM109 was used for plasmid amplification. The pBBR1MCS-5 plasmid, which contains the gentamicin resistance marker, was used for promoter strength characterization and gene overexpression.

### 2.2 Culture conditions

*E. adhaerens* HY-1 was grown on Luria–Bertani (LB) agar plates or in LB medium at 30 °C. Shaking flask studies were performed in complex media containing 100 g/L beet molasses, 0.7 g/L (NH_4_)_2_SO_4_, 0.02 g/L MnSO_4_, 0.02 g/L ZnSO_4_·7H_2_O, 1.5 g/L MgSO_4_·7H_2_O, 1.8 g/L (NH_4_)_2_HPO_4_, 0.02 g/L CoCl_2_, 0.01 g/L 5,6-dimethylbenzimidazole (DMBI), and 0.2 g/L emulsified silicone oil (pH = 7.3–7.4).

Fermentation medium contained 85 g/L maltose, 15 g/L betaine, 1.0 g/L KH_2_PO_4_, 0.1 g/L ZnSO_4_·7H_2_O, 0.084 g/L CoCl_2_, 0.07 g/L DMBI, 0.83 g/L FeCl_3_, 0.18 g/L glycerophosphate, 50 g/L corn syrup, 0.05 g/L MgCl_2_, 1.3 g/L MgSO_4_, 1.3 g/L (NH_4_)_2_SO_4_, 0.63 g/L urea, and 1.0 g/L CaCO_3_ (pH = 7.5–7.8).

Feeding medium contained 500 g/L glucose, 240 g/L betaine, 0.4 g/L DMBI, and 0.4 g/L CoCl_2_. Appropriate antibiotics were added to media: kanamycin (50 μg/mL) and gentamicin (50 μg/mL). Batch fermentations were performed in 250 mL shaking flasks containing 30 mL culture medium at 220 rpm at 30 °C on a reciprocal shaker (Zhichu, Shanghai, China). For fed-batch fermentations, the feeding medium was intermittently fed at different fermentation time (1 mL, 1 mL, and 2 mL were fed on days 3, 4, and 5, respectively).

### 2.3 Genome analysis of *E. adhaerens* HY-1

*E. adhaerens* HY-1 genomic DNA was extracted using Sangon Biotech Bacterial DNA kits (Shanghai, China). Genome sequencing was performed by Genewiz (Suzhou, China). *E. adhaerens* HY-1 genome annotation was completed using the RAST (https://rast.nmpdr.org/) website. The genomic data of the *E. adhaerens* HY-1 has been submitted to a sharing platform National Microbiology Data Center (NMDC, https://nmdc.cn/) and the number was NMDCN00010QE. Using the Kyoto Encyclopedia of Genes and Genomes (KEGG) database (https://www.genome.jp/kegg/), genes related to cobalamin synthesis in *E. adhaerens* HY-1 were obtained and identified. Gene sequences are shown (Table S2).

### 2.4 RNA-Seq of *E. adhaerens* HY-1

*E. adhaerens* HY-1 was grown in 30 mL fermentation medium, with samples removed daily for 2–7 days. Cells were washed three times in phosphate buffered saline (PBS, pH = 7.2) and RNA-Seq performed on samples by Genewiz (Suzhou, China) using the HiSeq 2500 platform. Based on RNA-Seq analyses, the Fragments Per Kilobase of transcript per Million mapped reads (FPKM) value of each gene was generated. According to the selection criterion, that is the FPKM values of genes were relative stable at the sampled points in the strain growth period and the average FPKM values of strain growth period were relative high. For our analyses, we selected 65 genes with the FPKM values spanning from 12.86 to 976159.7 and the average FPKM values of growth period in the 2225.6–296,023.2 range. (Table S3).

### 2.5 DNA manipulation

All study primers are listed (Supplementary Table S4). PCR and DNA ligations were performed according to manufacturer's instructions. All polymerase chain reaction (PCR) products were amplified by Phanta Max Master DNA Polymerase (Vezyme, Nanjing, China). DNA ligations were performed using the Ready-to-Use Seamless Cloning kit (Sangon, Shanghai, China) and different fragments were connected by 25 base pair (bp) overlaps. *E. coli* JM109 was then transformed with plasmids by heat-shock and *E. adhaerens* HY-1 via electro-transformation [23].

The P_*dnak*_ promoter was PCR-amplified using the primer pair, Pdnak-F/Pdnak-R and *E. adhaerens* HY-1 genomic DNA as template. Cobalamin metabolic pathway genes were PCR-amplified from *E. adhaerens* HY-1 genomic DNA. The linearized vector *RpBBR* was amplified using pBBR1MCS-5 as template, and RpBBR-F/RpBBR-R as primers. Fragments were ligated to generate the overexpression plasmid.

*mCherry* was amplified using mCherry-F/mCherry-R primers. Different promoters, *mCherry*, and the linearized *RpBBR* vector were then ligated to form a plasmid which was used to characterize promoter strength (Fig. 3A). The primers used to construct plasmids that characterize the strength of promoters is shown (Table S5). Promoters with different strength were combined with *CobSV*, *CobQ*, and *CobW* to generate the combined expression plasmid, pBBR-Pro-*CobSV*-Pro-*CobQ*-Pro-*CobW*. Finally, plasmids were verified by sequencing.

### 2.6 Genetic manipulation of *E. adhaerens* HY-1

*E. adhaerens* was cultured in LB medium to logarithmic growth phase, washed with sterile water and 10% glycerol, and then prepared into competent form, which can be stored in a −80 °C refrigerator for use. After the plasmid was transferred into *E. adhaerens* by electric shock method (0.1 cm electroporation cup, voltage 1.8 kV), 1 mL of LB medium was added to recover for 4 h, and then centrifuged and spread on the plate for culture.

### 2.7 Measurement of fluorescence intensity

Recombinant *E. adhaerens* HY-1 strains expressing fluorescence (*mCherry*) plasmids were inoculated into 30 mL fermentation medium in 250 mL shaking flasks (approximately 10% volume). Cultures were grown at 30 °C at 220 rpm, with samples removed every 12 h and washed three times in PBS. *mCherry* fluorescence intensity (excitation wavelength, 580 nm; emission wavelength, 610 nm) and optical density (absorbance at 600 nm) were measured using a microplate Multi-Mode Reader (Cytation 3, BIOTEK, NJ, USA) at different growth stages. Fluorescence intensity levels were defined as relative fluorescence units (RFU) divided by cell density (RFU/OD_600_) [24]. Fluorescence images were taken by a fluorescence microscope (Ci-L, Nikon, Japan). The *E. adhaerens* HY-1 strain harboring P_*dnak*_ was used as a control for promoter screening.

### 2.8 Analysis of optical density and cobalamin concentration

Cell densities were monitored by measuring the optical density at 600 nm (OD_600_). Cobalamin was measured as follows: 400 μL fermentation broth was collected and 40 μL NaNO_2_ 12.5% (w/v) and 40 μL glacial acetic acid added. The mixture was boiled for 30 min and centrifuged at 14,000 rpm for 5 min. Then, 500 μL supernatant was removed and 300 μL ddH_2_O and 200 μL KH_2_PO_4_ added. The mixture was resolved on a reverse phase C-18 column (4.6 × 250 mm, 5 μm, Thermo, MA, USA) using high performance liquid chromatography (HPLC) (Waters MA, USA) operating at 38 °C monitored at 361 nm. The mobile phase consisted of 28% methanol run at 0.8 mL/min for 18 min.

### 2.9 Statistical analysis

All the fermentation processes were performed in triplicate and the results were presented as mean values. The experimental data was analyzed by Origin 2019b. *P* values and significant difference were analyzed with *T*-test (p ≤ 0.1 was considered significant difference marked with a "∗", p ≤ 0.05 was considered extremely significant difference marked with a "∗∗").

## 3 Results

### 3.1 Genome sequencing and metabolic pathway analysis

To predict and identify key genes affecting cobalamin synthesis, the *E. adhaerens* HY-1 genome was sequenced and annotated using RAST software. All cobalamin synthesis-related genes from *E. adhaerens* HY-1 were compared with the *E. adhaerens* Casida A genome in the National Center for Biotechnology Information database. Using this approach, the following key information was ascertained. Based on the metabolic *de novo* synthesis of cobalamin by *E. adhaerens* in the KEGG database and *E. adhaerens* HY-1 genome sequencing results, several cobalamin synthesis genes were identified (Table 1).

**Table 1:** *E. adhaerens* HY-1 cobalamin synthesis related genes.

Gene Name	Definition
*HemA*	5-Aminolevulinate synthase
*HemB*	Porphobilinogen synthase
*HemC*	Hydroxymethylbilane synthase
*HemD*	Uroporphyrinogen-Ⅲ synthase
*CobA*	Uroporphyrin-Ⅲ C-methyltransferase
*CysG*	Uroporphyrin-Ⅲ C-methyltransferase
*CobI*	Precorrin-2 C(20)-methyltransferase
*CobG*	Precorrin-3B synthase
*CobJ*	Precorrin-3B C17-methyltransferase
*CobM*	Precorrin-4C(11)-methyltransferase
*CobF*	Precorrin-6A synthase
*CobK*	Cobalt-precorrin-6A reductase
*CobL*	Precorrin-6Y methyltransferase
*CobH*	Precorrin-8X methylmutase
*CobB*	Cobyrinic acid a,c-diamide synthase
*CobN*	Cobaltochelatase CobN
*CobS*	Cobaltochelatase CobS
*CobT*	Cobaltochelatase CobY
*CobR*	Cob(II)yrinic acid a,c-diamide reductase
*CobO*	Cob(I)alamin adenosyltransferase
*CobQ*	Adenosylcobyric acid synthase
*CobC*	Adenosylcobinamide-phosphate synthase
*CobD*	Adenosylcobinamide-phosphate synthase
*CobPU*	Adenosylcobinamide kinase
*CobSV*	Adenosylcobinamide-GDP ribazoletransferase
*BluB*	5,6-dimethylbenzimidazole synthase
*MetK*	S-adenosylmethionine synthetase
*CobW*	Cobalamin biosynthetic protein

### 3.2 Determining rate-limiting steps in the cobalamin synthesis pathway

**Fig. 2:**
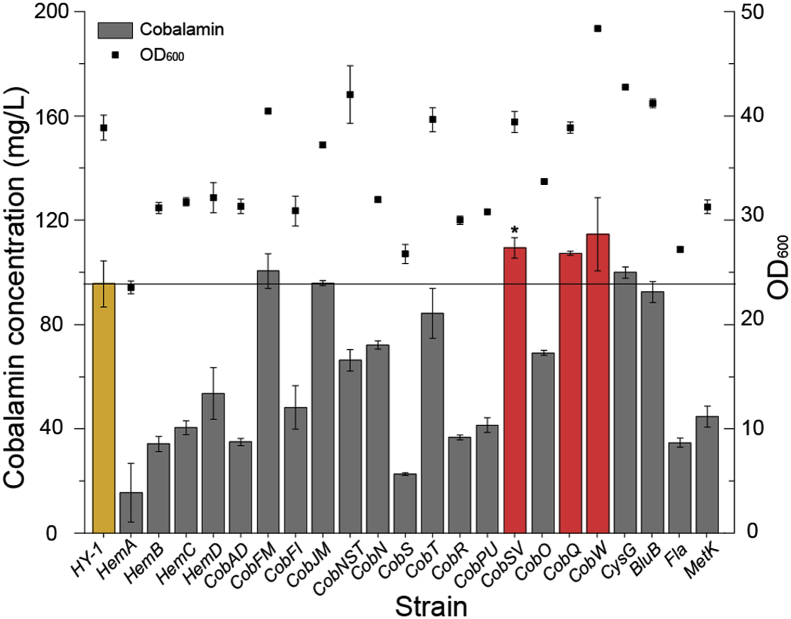
**Cobalamin production in overexpressing strains using the P**_***dnak***_**promoter.** The fermentation broths were sampled at the end of fermentation process (7.5 d) and the results were presented as mean values of the triplicate. The yellow bar represents the *E. adhaerens* HY-1 strain. The red bars represent strains where cobalamin production exceeded 10% of the control strain. Overexpression of *CobSV*, *CobQ*, and *CobW* with P_*dnak*_ enhanced cobalamin production by 14.4%, 12.2% and 19.9%, reaching 109.4 mg/L, 107.3 mg/L and 114.6 mg/L, respectively. The significant difference about cobalamin production in overexpressing strains with P_*dnak*_ promoter compared to that of wild-type strain HY-1 was analyzed with *T*-test, *p* ≤ 0.1 was considered significant difference marked with a "∗". *CobAD* means that the strain is overexpressed the gene cluster *CobA*-*CobD* (*CobA*, *CobB*, *CobC*, and *CobD*). CobFM means that the strain is overexpressed as the gene cluster *CobF*-*CobM* (*CobF*, *CobG*, *CobH*, *CobI*, *CobJ*, *CobK*, *CobL*, and *CobM*). CobFI means that the strain is overexpressed as the gene cluster *CobF*-*CobI* (*CobF*, *CobG*, *CobH*, and *CobI*). CobJM means that the strain is overexpressed as the gene cluster *CobJ*-*CobM* (*CobJ*, *CobK*, *CobL*, and *CobM*).

To realize the expression of exogenous plasmid in *E. adhaerens* HY-1, whose tolerance to different antibiotics was investigated. Results showed that the *E. adhaerens* HY-1 is sensitive to gentamicin, cephalosporin, chloramphenicol, tetracycline, and ampicillin (Table 2). It shows that PBBR1MCS-5 can self-replicate in *E. adhaerens* and can improve the drug resistance of *E. adhaerens*. To determine the rate-limiting steps and the influence of pathway genes on cobalamin synthesis, all relevant genes were overexpressed. To prevent gene overexpression burdening bacterial growth, a reported promoter P_*dnak*_ was used for gene expression [25]. Accumulated cobalamin was then investigated and verified by fermentation in shaking flasks. All the engineered strains were adjusted to same initial OD_600_ and were cultivated in the same conditions. The fermentation broths of the end of fermentation process (7.5 d) were used for comparative analysis. Three genes, *CobSV*, *CobQ*, and *CobW* were identified as the most efficient ones for enhancing cobalamin synthesis. Overexpression of *CobSV*, *CobQ*, and *CobW* with P_*dnak*_ enhanced the production of cobalamin by 14.4%, 12.2% and 19.9%, reaching 109.4 mg/L, 107.3 mg/L and 114.6 mg/L, respectively (Fig. 2). Additionally, a phenomenon was emerged from the graph that there seems to be a correlation between the cobalamin accumulation and OD_600_ of strains. But the specific mechanisms were needed to further investigate.

**Table 2:** *E. adhaerens* HY-1 resistance to different antibiotics.

Antibiotics	Concentration (μg/mL)	Resistance
Bleomycin	100	**+**
Gentamicin	50	**-**
Streptomycin	50	**+**
Kanamycin	50	**+**
Cephalosporin	100	**-**
Chloramphenicol	50	**-**
Tetracycline	50	**-**
Ampicillin	50	**-**

∗ “+” = positive resistance, “-” = negative resistance.

### 3.3 The identification of promoter fragments based on RNA-Seq

To understand gene transcriptional levels and promoter transcriptional intensities in *E. adhaerens* HY-1, transcriptome sequencing at different growth stages was performed. In total, we identified the expression levels of 6908 genes. It was worth noting that FPKM values of some genes varied greatly at different growth stages, with FPKM = 0 for some genes. Based on these observations, only genes with relatively stable, high expression levels were selected. Also, based on the relative position of the promoter and gene in the prokaryotic genome, selected promoter lengths ranged between 200 bp and 500 bp (Table S6). FPKM values of the selected 65 native promoters during the strain growth period were presented in Table S3.

**Fig. 3:**
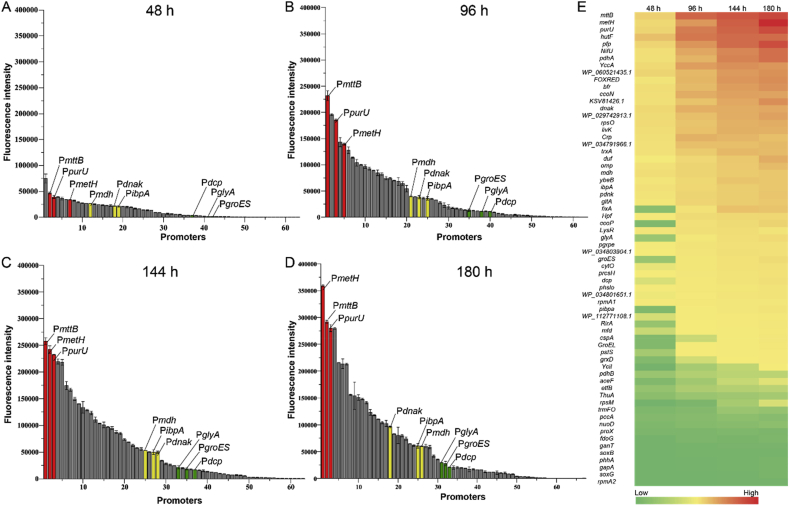
**Fluorescence intensity of 65 promoters at different *E. adhaerens* HY-1 growth periods**. The fermentation was performed in triplicate and the results were presented as mean values. **(A**–**D)** The red bar represents the strong promoter, P_*metH*_, the yellow bar represents the medium-strong promoter, P_*dnak*_, and the green bar represents the weak promoter, P_*groES*_. **(A)** Fluorescence levels in early log phase (48 h). **(B)** Fluorescence levels in medium log phase (96 h). **(C)** Fluorescence levels in the post-log phase (144 h). **(D)** Fluorescence levels at stationary phase (180 h). **(E)** Heatmap of promoter fluorescence intensity at different growth periods. Red indicates strong fluorescence intensity while green indicates weak fluorescence intensity.

To characterize promoter strength, the *mCherry* gene reporter and the shuttle plasmid, pBBR1MCS-5 were used for characterization. The promoter strength was divided based on the reported table medium strength promoter P_*dnak*_. Promoters resulting in high fluorescence intensity than that of P_*dnak*_ were divided as strong group, the rest were divided into group of medium-strength group and weak group according to the proximity to P_*dnak*_. *E. adhaerens* HY-1 expressing pBBR-P_*dnak*_-*mCherry* displayed red fluorescence under fluorescence microscopy. To determine the expression intensity of each promoter at different growth times, we tested early log (48 h), middle log (96 h), post log (144 h), and stationary phases (180 h) (Fig. 3). At early log phase, when compared with the promoter P_*dnak*_, expression intensity of most promoters were weaker (Fig. 3A). In the stable phase, fluorescence levels were stable and high. The highest fluorescing promoter, P_*metH*_ (RFU/OD_600_ = 358,876) was 5.8-fold higher than P_*dnak*_ (RFU/OD_600_ = 61,560), and 12.8-fold higher than the weak promoter, P_*groES*_ (RFU/OD_600_ = 28,005) (Fig. 3D). By analyzing the fluorescence microscopic image of three strain that respectively expressed *mCherry* by weak promoter (P_*glyA*_), medium-strong promoter (P_*dnak*_) and a strong promoter (P_*purU*_), the result was consistent with the measurement of fluorescence intensity (Fig. S1).

### 3.4 The combined expression effects of key genes on cobalamin production

**Fig. 4:**
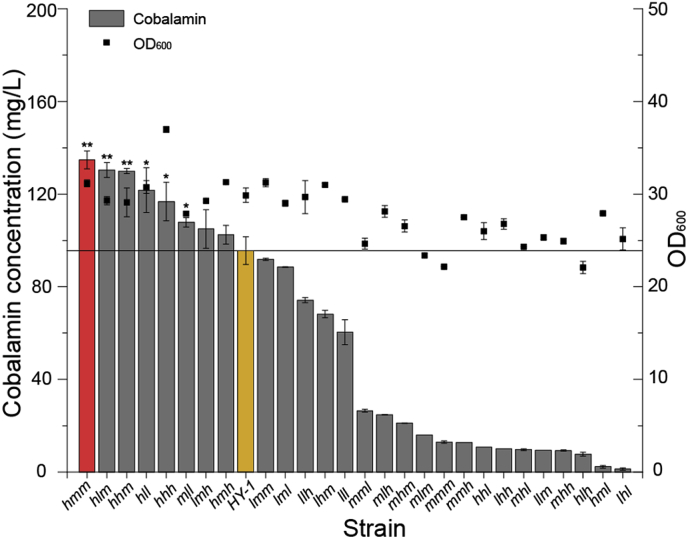
**The combined expression of different strength promoters and genes.** Different strains were constructed by combining *CobSV*, *CobQ*, *CobW* with different strength promoters, including three strong promoters (P_*metH*_, P_*mttB*_ and P_*purU*_), three medium-strong promoters (P_*dnak*_, P_*ibpA*_ and P_*mdh*_) and three weak promoters (P_*glyA*_, P_*groES*_ and P_*dcp*_). Fermentation broths were sampled at 7.5 d. The fermentation was performed in triplicate and the results were presented as mean values. The significant difference about cobalamin production of the positive engineered strains compared to that of wild-type strain HY-1 was analyzed with *T*-test, *p* ≤ 0.1 was considered significant difference marked with a "∗" and p ≤ 0.05 was considered extremely significant difference marked with a "∗∗". The yellow bar represents the *E. adhaerens* HY-1 strain. The red bar indicates the strain with the best combinatorial expression effects (*hmm*). The genotypes of different strains are shown in Table S1.

From our promoter strength and rate-limiting studies, different strength promoters and the three key genes, *CobSV*, *CobQ,* and *CobW* were combined for overexpression analyses. This generated 27 different combinations that were expressed in *E. adhaerens* HY-1 using the pBBR1MCS-5 plasmid (Table S1 and Fig. 4). The *hmm* strain (genotype: P_*metH*_-*CobSV*-P_*ibpA*_-*CobQ*-P_*mdh*_-*CobW*) was the most effective in accumulating cobalamin at 134.8 mg/L; this was 41.0% higher than the control strain (95.6 mg/L). It was showed that the combination of strong promoters with *CobSV* could generally yield better results. However, despites the *CobSV* was expressed by strong promoter, the strains *hhl*, *hlh* and *hml* just accumulated a small amount of cobalamin. Additionally, the cobalamin produced by strain *hhh*, in which the three key genes were all expressed by strong-promoters, was lower than those of the strain *hmm* and *hlm*, in which some genes were expressed by relatively weak promoters. These data indicated that *CobSV* was essential for cobalamin synthesis, and the performance of its function needed to well balancing expression of *CobQ* and *CobW*.

### 3.5 Optimizing the fermentation of combined strains

**Fig. 5:**
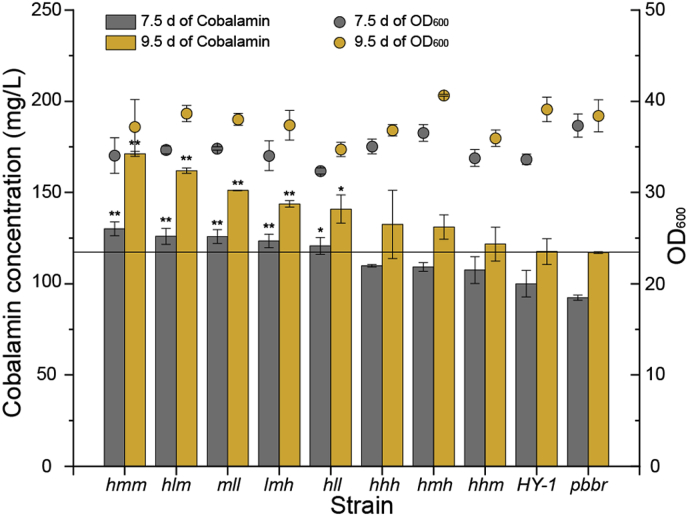
**Cobalamin production using fed-batch fermentation.** The original *E. adhaerens* HY-1 strain and the *pbbr* strain possessing plasmid pBBR1MCS-5 were used as controls. The fermentation was performed in triplicate and the results were presented as mean values. The significant difference about cobalamin production of the engineered strains at 7.5 d and 9.5 d compared to that of wild-type strain HY-1 was respectively analyzed with *T*-test, *p* ≤ 0.1 was considered significant difference marked with a "∗" and p ≤ 0.05 was considered extremely significant difference marked with a "∗∗". The gray bar (circle) represents that the fermentation broths samples at 7.5 d. The yellow bar (circle) represents that fermentation broth samples at 9.5 d. The bars present the cobalamin concentration, while the circles presents the OD_600_.

The eight engineered strains obtained above with positive effects on cobalamin production were further investigated using the fed-batch fermentation. Medium was fed on days 3–5 and cobalamin production detected in samples at 7.5 and 9.5 days. The original HY-1 strain and the *pbbr* strain carrying pBBR1MCS-5 served as controls. Expression data are shown (Fig. 5). When compared with the original HY-1 strain, recombinant cobalamin production levels were all increased. When fermentation continued for 7.5 days, the highest production level in the *hmm* strain was 130.1 mg/L, 30.1% higher than the original HY-1 strain. When fermentation continued for 9.5 days, cobalamin production was further enhanced to 171.2 mg/L, which was 45.6% higher than the original HY-1 strain.

## 4 Discussion

The *E. adhaerens* HY-1 strain *de novo* synthesizes cobalamin and has been used for cobalamin production in industry. To further understand strain characteristics, genome sequencing and annotation studies were performed. Genes related to cobalamin synthesis in *E. adhaerens* HY-1 were analyzed. We showed that cobalamin synthesis required the DMBI precursor for production [26]. Three key genes, *CobSV*, *CobQ*, and *CobW* were identified by gene overexpression studies with the promoter P_*dnak*_, and displayed considerable influences on cobalamin synthesis. Then, 65 promoters with different gradient strengths were identified by the transcriptome sequencing of cells at different growth stages. Using combinatorial overexpression of the obtained key three genes with different strength promoters, cobalamin levels reached 134.8 mg/L in shaking flasks, which were 41.0% higher than the original HY-1 strain. Finally, cobalamin production was further enhanced to 171.2 mg/L using a fed-batch fermentation strategy.

It was previously reported the cobalamin metabolic pathway involved more than 30 genes [18]. However, several genes and feedback regulatory mechanisms were not fully characterized, making cobalamin analysis and optimization extremely difficult [11,27,28]. Currently, cobalamin producing strains in the literature mainly comprise *E. adhaerens*, *Sinorhizobium meliloti* and *P. freudenreichii* [[29], [30], [31]]. *S. meliloti* belongs to the same species as *E. adhaerens* and aerobically synthesizes cobalamin [32]. However, restricted by the genetic operating systems, obtaining the high performance strain is mainly based on the random mutagenesis [33]. *P. freudenreichii* is reported to anaerobically synthesize cobalamin [34], but cell growth requires oxygen, and thus oxygen at early growth stages would be limited at later growth stages [35]. For cobalamin production in *E. adhaerens*, few reports are available. In this study, based on rate-limiting step analyses and the optimization of key gene expression, cobalamin production was enhanced to 171.2 mg/L using a fed-batch fermentation strategy. Currently, this is the highest cobalamin titer produced by *E. adhaerens*.

Unlike most typical model microorganisms, limited *E. adhaerens* synthetic biology tools were available for study. In previous work, the genome sequence of *E. adhaerens* OV14 was analyzed and the strain was reported for usage as a vector for plant plasmid transformations [36]. In addition, it was found that plasmid could be transferred into *E. adhaerens* by quick freezing with liquid nitrogen [37]. Nonetheless, genetic manipulation tools for *E. adhaerens* were lacking which meant metabolic regulation strategies were impossible to implement. The pBBR1MCS-5 plasmid could be successfully transformed into *E. adhaerens* HY-1 cells and laid the foundations for the genetic manipulations of the industrial producer. Additionally, the gradient-strength promoters identified from our transcriptome analyses now form a valuable reference database for future *E. adhaerens* research.

The cobalamin synthesis pathway is subjected to feedback inhibition by various regulatory mechanisms in strains that accumulate cobalamin. It was previously noted that cobalamin synthesis was regulated by highly conserved RNA structures, riboswitches [38], which responded to cobalamin expression [39]. Upon cobalamin accumulation in cells, strong inhibitory effects may impact gene expression associated with cobalamin synthesis [19]. In addition, riboswitches appear to exert transcriptional level effects on most cobalamin synthesis genes [[40], [41], [42]]. In this study, three key cobalamin synthesis genes were identified. The function of *CobW* remains unclear, but it was a significant player in cobalamin synthesis. The last step of cobalamin synthesis involves the conversion of adenosylcobinamide-GDP to adenosylcobalamin, which was encoded by *CobSV* and identified as the rate-limiting step. This has not been previously reported. *CobQ*, which encodes adenosylcobyric acid synthase, was identified as promoting adenosylcobyric acid production. Besides, overexpression of upstream genes in the cobalamin synthesis pathway, such as *HemA*, *HemB*, *HemC* and *HemD* led to dramatically decrease of cobalamin production. It was speculated that the potential accumulation of intermedium metabolites caused by overexpressing these upstream genes could disrupt the metabolism of cobalamin. Since the lack of standards for these complicated intermediates, the specific mechanism should be further investigated.

In conclusion, a high titer cobalamin-producing strain was identified via promoter screening, rate-limiting step analyses, and combined gene expression. Cobalamin production levels reached 171.2 mg/L, which were increased by 45.6% when compared to the original *E. adhaerens* HY-1 strain. In the future, other strategies could be implemented to further enhance cobalamin synthesis. For example, the DMBI precursor is required for cobalamin synthesis but it exerts inhibitory effects on bacterial growth [26]. Thus, a balanced approach could be adopted between bacterial growth and cobalamin accumulation by optimizing the DMBI synthesis pathway [43]. Because cobalamin synthesis requires ATP, it may be possible to improve energy supplies and optimize cofactors to balance metabolic flow [44]. In addition, inhibiting competitive pathways using CRISPR and CRISPRi could be investigated [[45], [46], [47]] as well as enhancing strain tolerance to precursors using adaptive evolution.

## Author contributions

S. X., and Z. X. designed the study and wrote the manuscript. J. Z., and W. Z critically revised the manuscript. Z. X., S. Y., and Y. Z. performed the experiments and analyzed the results. J. Z., S. X., and W. Z. designed and supervised the project. All authors discussed the results and commented on the manuscript.

## Declaration of competing interest

The authors declare no competing interests.
