# Supplementary material for: Enhanced cobalamin biosynthesis in Ensifer adhaerens by regulation of key genes with gradient promoters
Source: Synth Syst Biotechnol. 2022 May 4;7(3):941–8. doi: 10.1016/j.synbio.2022.04.012 (PMC9157374; doi:10.1016/j.synbio.2022.04.012)
Supplement: Multimedia component 2 [file mmc2.docx]

# Enhanced cobalamin biosynthesis in *Ensifer adhaerens* by regulation of key genes with gradient promoters

Sha Xu^1,2,4^, Zhiqiang Xiao^1,2,3#^, Shiqin Yu^1,2,3^, Weizhu Zeng^2,3^, Yongming Zhu^5^, Jingwen Zhou^1,2,3,4*^

^1^ National Engineering Laboratory for Cereal Fermentation Technology, Jiangnan University, 1800 Lihu Road, Wuxi, Jiangsu 214122, China.

^2^ School of Biotechnology and Key Laboratory of Industrial Biotechnology, Ministry of Education, Jiangnan University, 1800 Lihu Road, Wuxi, Jiangsu 214122, China;

^3^ Science Center for Future Foods, Jiangnan University, 1800 Lihu Road, Wuxi, Jiangsu 214122, China.

^4^ Jiangsu Provisional Research Center for Bioactive Product Processing Technology, Jiangnan University, 1800 Lihu Road, Wuxi, Jiangsu 214122, China;

^5^ Hunan Hongying Biotechnology Co. Ltd., 10 Hongying Road, Jinshi, Hunan 415400, China.

* Corresponding author: Jingwen Zhou

Mailing address: Science Center for Future Foods, Jiangnan University, 1800 Lihu Road, Wuxi, Jiangsu 214122, China

Phone: +86-510-85914371, Fax: +86-510-85914371

E-mail: zhoujw1982@jiangnan.edu.cn.

^#^ This author contributed equally to this work.

**
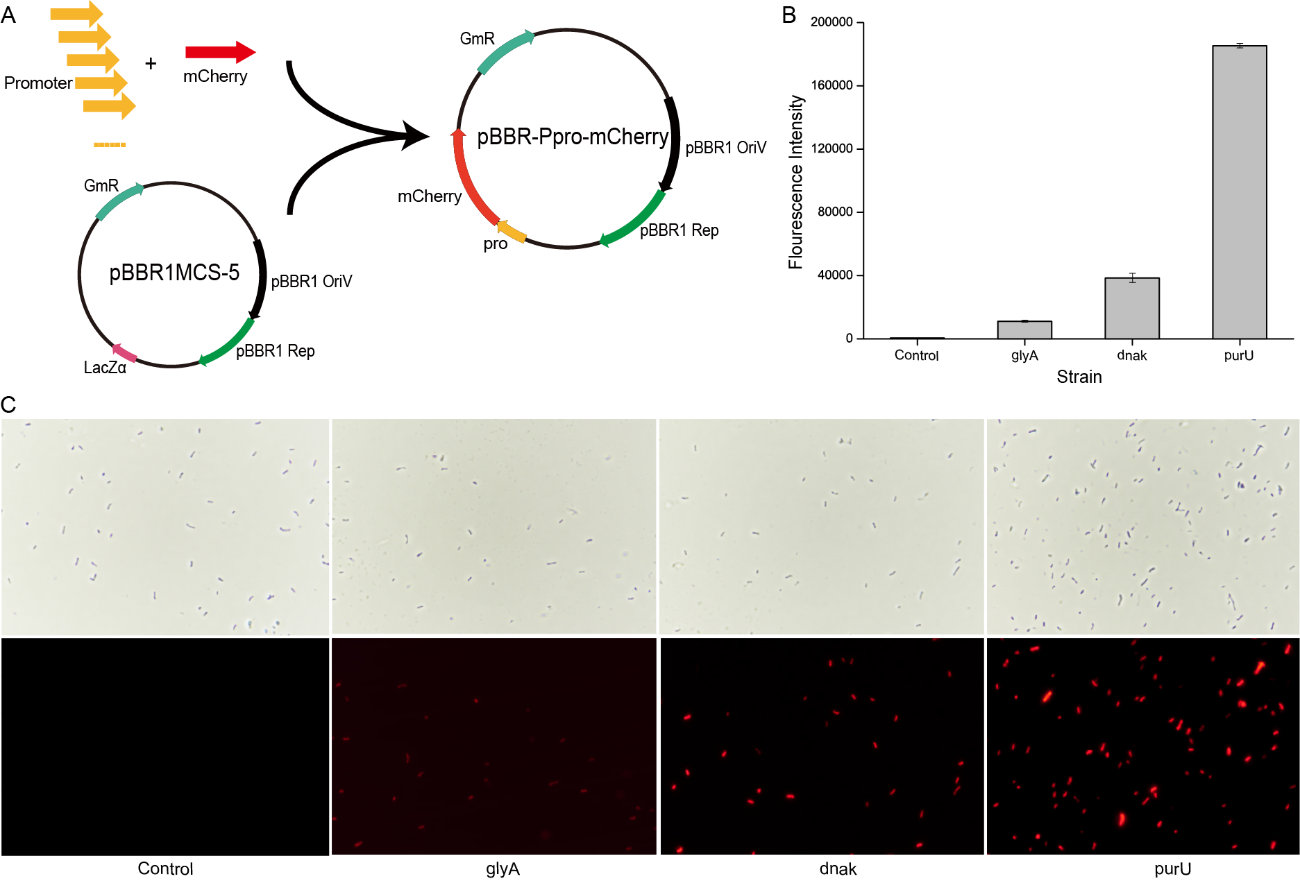
**

## Figure S1 Construction of promoter screening plasmid and the picture of different promoters under microscope

The *mCherry* was as gene reporter and the shuttle plasmid pBBR1MCS-5 was applied for gene expression in the *E. adhaerens* HY-1. The three strain were expressed *mCherry* by weak promoter (P*_glyA_*), medium-strong promoter (P*_dnak_*) and strong promoter (P*_purU_*), respectively. The original *E. adhaerens* HY-1 strain was as the control. (A) Construction process of promoter screening plasmid. (B) Fluorescence intensity of selected promoters. (C) Fluorescence of strain under the fluorescence microscope.
